# USAF Characteristic *K* Approach: A Robust Tool for Predicting Fatigue Crack Growth under Various Underload Spectra

**DOI:** 10.3390/ma17133303

**Published:** 2024-07-04

**Authors:** Kushagra Tiwari, Alankar Alankar, R. K. Singh Raman, Rhys Jones

**Affiliations:** 1 Department of Mechanical Engineering, Indian Institute of Technology Bombay, Mumbai 400076, India; kushagra.tiwari@monash.edu (K.T.); alankar.alankar@iitb.ac.in (A.A.); 2 Department of Mechanical and Aerospace Engineering, Monash University, Clayton, VIC 3800, Australia; rhys.jones@monash.edu; 3 IITB-Monash Research Academy, Indian Institute of Technology Bombay, Mumbai 400076, India; 4 ARC Industrial Transformation Training Centre on Surface Engineering for Advanced Materials, Faculty of Science, Engineering and Technology, Swinburne University of Technology, John Street, Hawthorn, VIC 3122, Australia

**Keywords:** durability, USAF Characteristic *K*, Hartman–Schijve, underloads, AM

## Abstract

This paper forms part of an ongoing investigation into the tools required in linear elastic fracture mechanics (LEFM) for evaluating the durability of components designed for limited life replacement. In this study, we demonstrate that the USAF ‘Characteristic *K*’ method, when combined with the Hartman–Schijve adaptation of the NASGRO crack growth formula, can predict the impact of underloads on the propagation of small cracks in aluminum alloy AA7050-T7451 with reasonable accuracy. The published *da/dN* versus Δ*K* small crack growth curves associated with five specific underload spectra are examined. It is found that, in each case, there is reasonably good agreement between the predicted and the measured curves. To the best of the author’s knowledge, this paper is the first to highlight the ability of the USAF Characteristic *K* approach, when coupled with the Hartman–Schijve equation, to reasonably accurately predict the growth of small cracks subjected to a range of underload spectra.

## 1. Introduction

The 2019 memo [1] mandated the implementation of additive manufacturing (AM) practices across the United States Department of Defense (DoD). US Army Directive 2019-29 [2] highlighted the transformative potential of additive manufacturing in battlefield logistics by enabling the immediate fabrication of parts close to where they are needed. USAF Structures Bulletin EZ-19-01 [3] identified one of the primary obstacles in the airworthiness certification of AM components as “accurate prediction of structural performance”, particularly in terms of DADT (durability and damage tolerance). An important feature of AM is that, as outlined in [1,2,3,4], it enables the production of structural components on demand. Although these components may have a shorter lifespan than originally designed, they are adequate to maintain operational capabilities until a traditionally manufactured replacement is available. However, according to Structures Bulletin EZ-19-01 and MIL-STD-1530Dc [5], evaluating the airworthiness of a part with limited life necessitates a durability analysis. By this, it is meant that an accurate prediction of the growth of small naturally occurring cracks is required [6,7,8] (Here the terminology “small crack” is as defined in the ASTM fatigue test standard ASTM E647-13a [9]). Consequently, one challenge facing the airworthiness certification of AM parts is the development and validation of analysis tools capable of predicting the durability of AM parts. The same challenge is faced for the airworthiness certification of cold spray repairs, alternatively referred to as supersonic particle deposition or SPD repairs and which involves repair using an additive metal deposition process, to military aircraft [10,11,12,13].

Within this framework, it is important to acknowledge the requirements set forth in MIL-STD-1530Dc [5] and noted in the US Joint Services Structural Guidelines [14], that the analyses should follow a building block approach. Furthermore, as delineated in the NASA Fracture Control Handbook NASA-HDBK-5010 [15], given the variability which can be associated with the *da*/*dN* versus Δ*K* curves (hereinafter referred to as ‘fatigue curves’) associated with both long and small cracks in both conventionally and additively manufactured materials [6,8,16,17,18,19], NASA-Handbook-5010 mandates that the certification analysis should use an upper bound (worst case) crack growth curve.

In this scenario, it is pertinent to highlight that, as shown in [20,21,22,23,24], underloads in a fatigue spectrum can accelerate the growth of long cracks. (Underloads are characterized as loading cycles where the minimum stress is considerably lower than that seen in the neighboring cycles.) Hence, to be consistent with the mandated building block approach to certification of limited life parts, it is important to be able to account for the effect of underloads on the growth of small naturally occurring cracks.

At this point in the discussion, it is important to acknowledge that the USAF Damage Tolerance Design Handbook [25,26] outlines this from a design perspective, and the growth of cracks in operational aircraft can often be determined by approximating the flight load spectra as a series of repeating constant amplitude load blocks. We will term this approach the USAF ‘Characteristic *K*’ approach. Examples of the use of various variants of the Characteristic *K* approach can be found in [6,7,17,27,28,29,30,31].

Consequently, it is noted that:

1. USAF MIL-STD-1530Dc stipulates that the airworthiness certification of military aircraft necessitates a durability analysis. This analysis should follow a building block approach, where the equation describing crack growth under constant amplitude loads must demonstrate its capability to handle more intricate, real-world load spectra;
2. Refs. [6,7,17,28,29] indicate that methods based on the Characteristic *K* approach may be appropriate for sustainment-related analyses;
3. Refs. [6,7,8,17,18,32,33,34,35,36,37,38,39,40,41] show that the Hartman–Schijve equation for crack growth is frequently applicable for forecasting the durability of materials manufactured both conventionally and through AM processes;
4. Refs. [12,17] demonstrates that the Hartman–Schijve equation for crack growth is often effective in estimating the durability of cold spray repairs, addressing both simulated corrosion damage and intergranular corrosion around fastener holes;
5. Refs. [42,43] indicate that the Hartman–Schijve equation for crack growth is frequently applicable for modeling the durability of parts produced via cold spray additive manufacturing;
6. While the 2014 review paper [7] notes, that several crack growth equations are typically employed for assessing the damage tolerance of conventionally manufactured metals, their effectiveness in accurately forecasting the growth of small, naturally occurring cracks in AM components and cold spray repairs has not yet been confirmed.

This paper primarily aims to investigate if the USAF Characteristic *K* approach, when used in conjunction with the Hartman–Schijve variant of the NASGRO crack growth equation [7], can be used to predict the *da*/*dN* versus Δ*K* small crack growth curves associated with AA7050-T7451 alloy specimens subjected to the different underload spectra that were studied in [44]. These test cases were chosen since the aluminium alloy AA7050-T7451 is widely used in the Boeing F/A-18E/F Super Hornet, the Boeing EA-G Growler, and in the Lockheed Martin F-35 Lightning II. The outcome of this investigation is that it is found that the fatigue curves seen in the various underload tests reported in [44] can be reasonably well predicted using the USAF Characteristic *K* approach. *Here it is worth mentioning that, as far as the authors are aware, there has been no prior study establishing the ability of the Characteristic K approach to reasonably accurately predict the growth of small cracks under various underload spectra.*

## 2. The USAF Characteristic *K* Approach

The USAF Damage Tolerant Design Handbook [25,26] explains that the damage tolerance analysis of many real-world engineering problems that involve complex variable amplitude load spectra can be simplified by expressing the load spectra as a sequence of recurring constant amplitude load blocks. In such instances, [25,26] suggest that crack growth can be computed using an equation of the form

(1) $$\frac{da}{dB}=D{\left(K_{char}\right)}^{m}$$

Here, *da*/*dB* represents the increase in length of the crack with each load block (*B*), with *m* and *D* being constants. *K_char_* denotes a characteristic stress intensity factor value that is associated with the load block, and is given as

(2) $$K_{char}=\sigma_{char}\beta \sqrt{\pi a}.$$

Here *σ_char_* is the corresponding characteristic stress in the load block and *β* is the beta (geometry) factor associated with the problem being analyzed. Two of the most commonly used forms for the characteristic stresses are the maximum stress (*σ_max_*) and the root mean square (RMS) stress (*σ_rms_*) in the block [7,17,25,28]).

The version of the characteristic *K* variant of the Hartman–Schijve crack growth equation presented in [7] is:

(3) $$\frac{da}{dB}=D\frac{{\left(\Delta K_{char}-\Delta K_{char,thr}\right)}^{p}}{{\left(1-\frac{K_{max}}{A}\right)}^{\frac{p}{2}}}$$

In this context, *K_max_* represents the peak value of the stress intensity factor within a specified load block, i.e.,

(4) $$K_{max}=\sigma_{max}\beta \sqrt{\pi a}.$$

where *σ_max_* denotes the highest stress within the load block, Δ*K_char_*_,*thr*_ acts as a term akin to a fatigue threshold, and A represents the cyclic fracture toughness. Setting Δ*K_char_* = Δ*K_rms_*, where

(5) $$K_{rms}=\sigma_{rms}\beta \sqrt{\pi a}.$$

Equation (3) reduces to

(6) $$\frac{da}{dB}=D\frac{{\left(\Delta K_{rms}-\Delta K_{rms,thr}\right)}^{p}}{{\left(1-\frac{K_{max}}{A}\right)}^{\frac{p}{2}}},$$

where Δ*K_rms_*_,*thr*_ is the corresponding fatigue threshold. As per [17], it is convenient to introduce term *f_rms_* to relate Δ*K_rms_* to *K_max_*, i.e.,

(7) $$\Delta K_{rms}=f_{rms}K_{max}.$$

The term *f_rms_* is calculated as

(8) $$f_{\mathrm{rms}}=\sqrt{\frac{\sum_{i=1}^{n}{\left(N_{i}\left(1-R_{i}\right)\right)}^{2}}{\sum_{i=1}^{n}N_{i}}},$$

where *N_i_* and *R_i_* denote the number of cycles and the corresponding *R* ratio of the *i*th load block, respectively, and *n* represents the total number of load blocks. As outlined in [7,8,12,17,36,39,40,41], when using this formulation to predict the worst-case (fastest) crack growth curve associated with the growth of small cracks, the threshold term Δ*K_rms_*_,*thr*_, or the term Δ*K_thr_* depending on if the Characteristic *K* approach is or is not used, should be set to a small value, typically in the range 0.1 to 0.3 MPa √m. (The fastest growing cracks in an airframe are commonly referred to as “lead cracks” [6,16,45,46]). Whilst, as noted above, this formulation has been used to accurately compute the growth of small cracks subject to a range of variable amplitude spectra, its applicability to spectra having underloads has not yet, to the best of the author’s knowledge, been studied.

Consequently, the ensuing question emerges: Can this equation (Equation (6)) be used for predicting crack growth from small naturally occurring cracks subjected to different underload spectra? This question will be addressed in the next section.

## 3. Fatigue Crack Growth under a Range of Underload Spectra

Field et al. [44] examined the impact of underload on small three-dimensional surface breaking cracks, with depths of approximately 0.1 mm, in 7050-T7451. The effect of underload magnitude, spacing and consecutive application on crack growth is discussed in detail. In the study by Field et al. [44], a total of six different loading spectra were studied. Each spectrum consisted of a number of load blocks. The nomenclature used by Field et al. [44] to describe each of these various spectra and the load blocks that made up each spectrum, followed the format Ry_1_ULy_2_Sy_3_, where *y*_1_ denotes the *R* ratio (of underloads), *y*_2_ indicates the consecutive underloads, and *y*_3_ represents the number of baseline cycles that had *R* ratio as 0.5 and were applied between each underload application. See [44] for more details. To help clarify this notation, a few examples are presented in Table 1.

It should also be noted that each of these repeated spectra were followed by (separated by) 500 cycles at *R* = 0.7. The maximum stress in each spectrum and load block was held constant at 240 MPa. The values of *f_rms_* associated with the different load blocks that made up the various spectra are given in Table 2.

The constants *D*, and *p* for 7050-T7451 were given in [18,40,41] as 7.0 × 10^−10^ and 2, respectively. For this specific material 7050-T7451, the fracture toughness was taken from the ASM Materials data sheet [47], namely *A* = 32 MPa √m. Consequently, following the approach outlined above, setting the fatigue threshold term (Δ$K_{rms,thr}$) related to the growth of small naturally occurring lead cracks as Δ$K_{rms,thr}$ = 0.1 MPa √m, we obtain:

(9) $$\frac{da}{dB}={\left(7.0\times 10\right)}^{-10}\frac{{\left(\Delta K_{rms}-0.1\right)}^{2}}{\left(1-\frac{K_{max}}{32}\right)}$$

**Figure 1:**
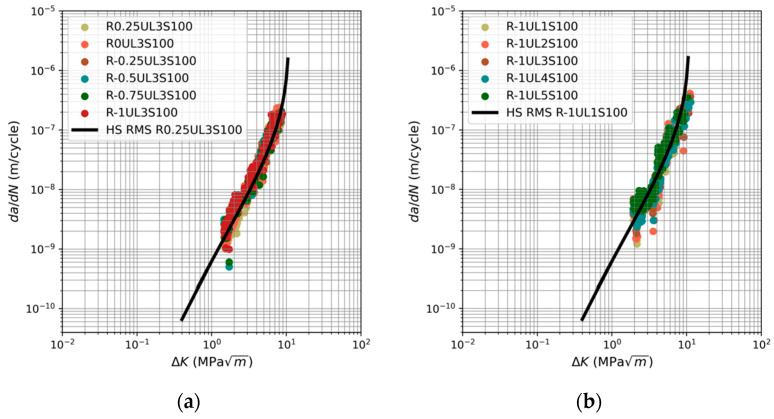

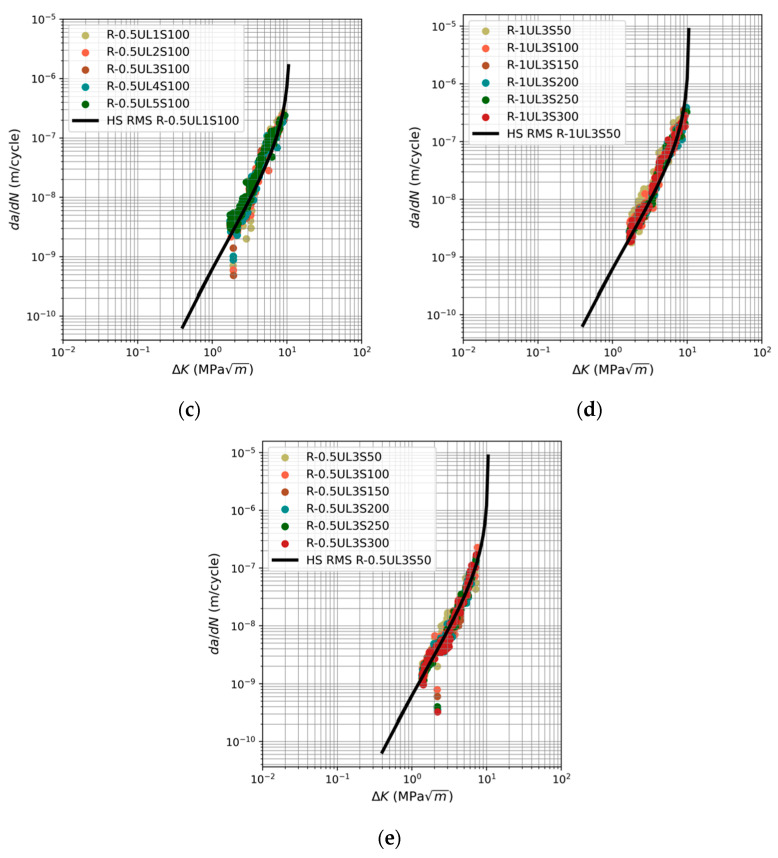
Fatigue crack growth predictions and the corresponding curve given in [44] with *A* = 32 MPa √m for load spectra: (**a**) R0.25:-1UL3S100, (**b**) R-1UL1:5S100, (**c**) R-0.5UL1:5S100, (**d**) R-1UL3S50:300, and (**e**) R-0.5UL3S50:300.

Equation (9) was subsequently employed to forecast the fatigue curves detailed in [44] for each spectrum analyzed. These predictions are also shown in Figure 1. This also shows the actual crack growth data (*da*/*dN* vs. Δ*K*) that is extracted from [44].

**Figure 2:**
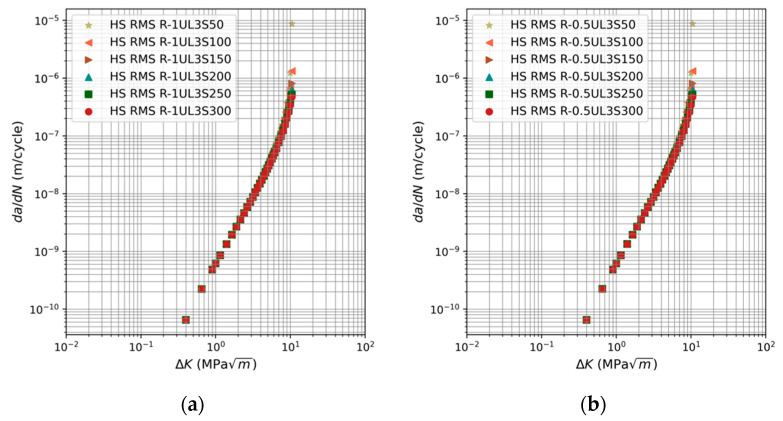
Plots of the various computed fatigue curves associated with spectra: (**a**) R-1UL3S50:300 and (**b**) R-0.5UL3S50:300.

Figure 1 indicates that the various fatigue curves given in [44] are generally predicted with good accuracy by the USAF Characteristic *K* method. It should also be noted that, whilst the objective of this study was not to fit the measured data, the coefficient of determination (*R*^2^) associated with the fits to the fastest data sets, i.e., the data shown in Figure 1e, is greater than 0.8. That said, it should be noted that each figure shows only one prediction. The similarity in term *f_rms_* values for a specific load spectrum results in predicted curves that are closely aligned. This phenomenon is clearly demonstrated in Figure 2, where the curves corresponding to spectra R-1UL3S50:300 and R-0.5UL3S50:300 are depicted respectively. Consequently, presenting just one prediction provides a clearer picture of the concordance between the predicted curves and the measured data.

**Figure 3:**
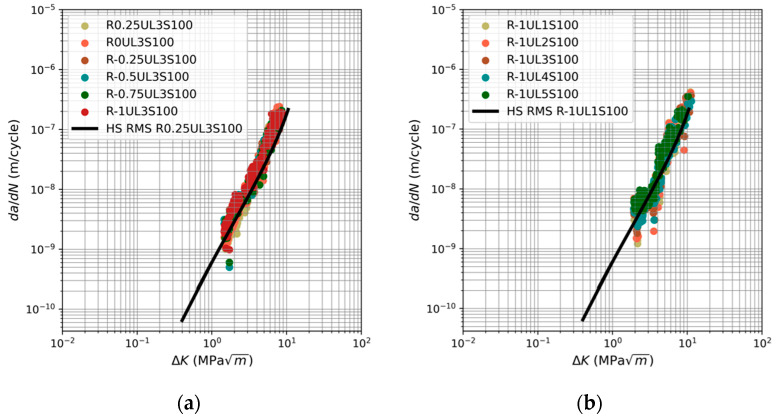

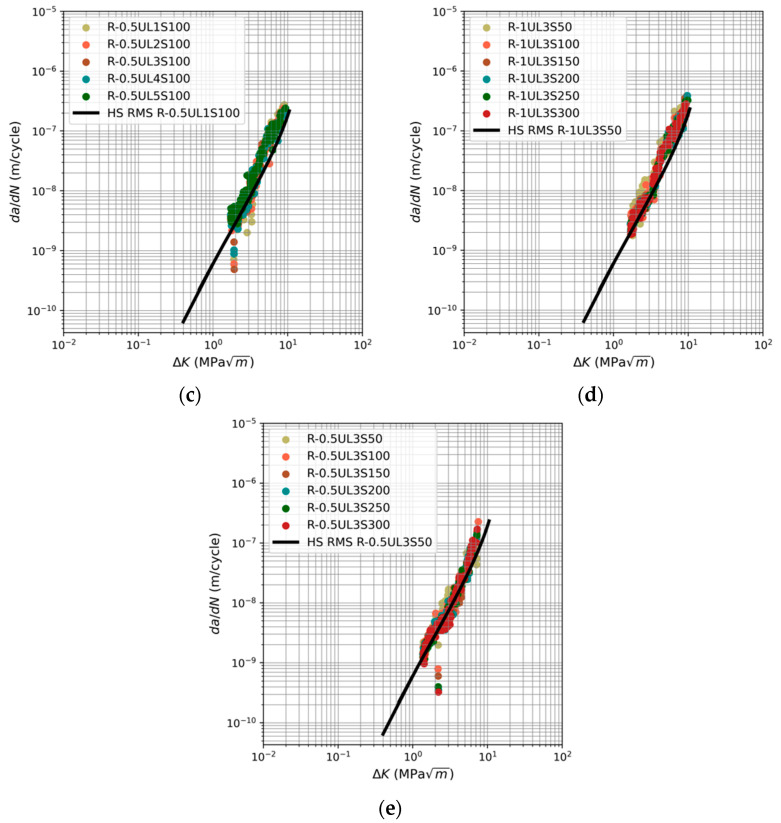
Fatigue crack growth predictions and the corresponding curve given in [44] with *A* = 47 MPa √m for load spectra: (**a**) R0.25:-1UL3S100, (**b**) R-1UL1:5S100, (**c**) R-0.5UL1:5S100, (**d**) R-1UL3S50:300, and (**e**) R-0.5UL3S50:300.

Whilst the consistency between the measured and the predicted fatigue curves is very good, the question emerges regarding how variations in the value of *A* influence the various calculated crack growth curves. To investigate this effect, the analyses were repeated using the value of *A* given in [40,41], namely *A* = 47 MPa √m. The consequent measured and computed curves are displayed in Figure 3. Here it is seen that, whilst using the value listed in the ASM data sheet gave excellent predictions, using a value of *A* = 47 MPa √m nevertheless yields reasonably good predictions. As such, it would appear that the sensitivity of the predicted curves to the value of *A* is not a particularly significant issue.

## 4. Conclusions

This paper forms part of an ongoing investigation into the linear elastic fracture mechanics (LEFM) tools needed to assess the durability of both limited life AM replacement parts and cold spray repairs. The USAF Damage Tolerance Design Handbook permits the application of the ‘Characteristic *K’* methodology in studies related to airworthiness certification. At the same time, MIL-STD-1530Dc requires a predictive capability. This study illustrates how the USAF Characteristic *K* method, when used in conjunction with the Hartman–Schijve version of the NASGRO crack growth equation, reasonably accurately predicts the impact of underloads on the growth of small cracks in the aluminium alloy AA7050-T7451. The specific test cases analysed were chosen since the aluminium alloy AA7050-T7451 is widely used in the Boeing F/A-18E/F Super Hornet, the Boeing EA-G Growler, and in the Lockheed Martin F-35 Lightning II. In this context, it should be noted that [44] reported that the underload spectra studied were deliberately constructed so as to investigate the influence of underload spacing, consecutive application, and magnitude on crack growth rates. The study demonstrates that the USAF Characteristic *K* method effectively predicts the fatigue crack growth rate across all five analyzed underload spectra. Furthermore, it was found that the sensitivity of the predicted curves to the value of parameter *A* is not a significant issue.

Based on the author’s knowledge, this paper represents the first instance of demonstrating the Characteristic *K* approach’s capacity to predicting the growth of small cracks under various underload spectra with reasonable accuracy. To further validate and solidify its effectiveness, future work will involve applying this approach to other materials subjected to different underload spectra, ultimately establishing it as a robust predictive tool.

## Figures and Tables

**Table 1:** Clarification of the nomenclature used in the various spectra.

Nomenclature of Load Spectra	Loading Pattern
R0.25UL3S100	3 cycles @ R = 0.25 + 100 cycles @ R = 0.5
R-1UL1S100	1 cycle @ R = −1.0 + 100 cycles @ R = 0.5
R-1UL3S50	3 cycles @ R = −1.0 + 50 cycles @ R = 0.5
R-0.5UL3S150	3 cycles @ R = −0.5 + 150 cycles @ R = 0.5

**Table 2:** Values of *f_rms_* associated with the different spectra.

Load Spectra		*f_rms_*
R0.25:-1UL3S100	R0.25UL3S100	0.345
	R0UL3S100	0.348
	R-0.25UL3S100	0.348
	R-0.5UL3S100	0.348
	R-0.75UL3S100	0.348
	R-1UL3S100	0.348
R-1UL1:5S100	R-1UL1S100	0.344
	R-1UL2S100	0.346
	R-1UL3S100	0.348
	R-1UL4S100	0.350
	R-1UL5S100	0.352
R-0.5UL1:5S100	R-0.5UL1S100	0.344
	R-0.5UL2S100	0.346
	R-0.5UL3S100	0.348
	R-0.5UL4S100	0.350
	R-0.5UL5S100	0.352
R-1UL3S50:300	R-1UL3S50	0.331
	R-1UL3S100	0.348
	R-1UL3S150	0.362
	R-1UL3S200	0.373
	R-1UL3S250	0.383
	R-1UL3S300	0.391
R-0.5UL3S50:300	R-0.5UL3S50	0.331
	R-0.5UL3S100	0.348
	R-0.5UL3S150	0.362
	R-0.5UL3S200	0.373
	R-0.5UL3S250	0.383
	R-0.5UL3S300	0.391

## Data Availability

The original contributions presented in the study are included in the article, further inquiries can be directed to the corresponding author.

## Nomenclature

*a*	crack length
*D,m*	constants in the Hartman–Schijve and Nasgro crack growth equations
*AM*	additive manufacturing
*da/dN*	rate of fatigue crack growth
*β*	beta (geometry) factor
*K*	stress intensity factor
*K_max_*	maximum stress intensity factor
*K_char_*	characteristic stress intensity factor
*ΔK_char_*	characteristic stress intensity factor range
*ΔK_char,thr_*	fatigue threshold associated with Δ*K_char_*
*K_rms_*	the rms value of stress intensity factor
*ΔK_rms_*	the rms value of stress intensity factor range
*ΔK_rms,thr_*	fatigue threshold associated with Δ*K_rms_*
*σ_char_*	characteristic stress
*σ_rms_*	rms value of stress
*A*	cyclic fracture toughness
DADT	durability and damage tolerance
LEFM	linear elastic fracture mechanics
NASA	North American Space Administration
DoD	Department of Defense
USAF	United States Air Force
